# Induction of Serum Amyloid A3 in Mouse Mammary Epithelial Cells Stimulated with Lipopolysaccharide and Lipoteichoic Acid

**DOI:** 10.3390/ani11061548

**Published:** 2021-05-25

**Authors:** Sato Kamiya, Kaori Shimizu, Ayaka Okada, Yasuo Inoshima

**Affiliations:** 1 Laboratory of Food and Environmental Hygiene, Cooperative Department of Veterinary Medicine, Faculty of Applied Biological Sciences, Gifu University, Gifu 501-1193, Japan; Nana.sa08to03.cl@gmail.com (S.K.); skaori@gifu-u.ac.jp (K.S.); okadaa@gifu-u.ac.jp (A.O.); 2 Education and Research Center for Food Animal Health, Gifu University (GeFAH), Gifu 501-1193, Japan; 3 Joint Graduate School of Veterinary Sciences, Gifu University, Gifu 501-1193, Japan; 4 The United Graduate School of Veterinary Sciences, Gifu University, Gifu 501-1193, Japan

**Keywords:** lipopolysaccharide, lipoteichoic acid, mouse mammary epithelial cells, serum amyloid A1, serum amyloid A3

## Abstract

**Simple Summary:**

Serum amyloid A (SAA) is an acute phase protein present in mammals and birds. Based on the amino acid sequence, SAA has been classified into isoforms SAA1–4 in mice. Previously, it was reported that after the stimulation with bacterial antigens, the expression of the *Saa3* mRNA was induced more strongly than that of the *Saa1* mRNA in mouse epithelia, including colonic and alveolar epithelial cells, indicating that SAA3 plays a role in the local response. However, the contribution of SAA3 to the local response in mouse mammary epithelium, where mastitis occurs due to bacterial infection, has not been completely determined yet. In this study, to clarify whether mouse SAA3 has a role in the defense against bacterial infection in mouse mammary epithelium, normal murine mammary gland (NMuMG) epithelial cells were stimulated with lipopolysaccharide (LPS) and lipoteichoic acid (LTA). LPS and LTA significantly enhanced mRNA expression level of the *Saa3* gene but not that of *Saa1*. Furthermore, LPS induced SAA3 protein expression more strongly than LTA. Our data indicate that SAA3 expression in mouse mammary epithelial cells was increased by the stimulation with bacterial antigens, suggesting that SAA3 is involved in the defense against bacterial infection in mouse mammary epithelium.

**Abstract:**

In this study, to establish whether serum amyloid A (SAA) 3 plays a role in the defense against bacterial infection in mouse mammary epithelium, normal murine mammary gland (NMuMG) epithelial cells were stimulated with lipopolysaccharide (LPS) and lipoteichoic acid (LTA). LPS and LTA significantly enhanced mRNA expression level of the *Saa3* gene, whereas no significant change was observed in the *Saa1* mRNA level. Furthermore, LPS induced SAA3 protein expression more strongly than LTA, whereas neither LPS nor LTA significantly affected SAA1 protein expression. These data indicate that the expression of SAA3 in mouse mammary epithelial cells was increased by the stimulation with bacterial antigens. SAA3 has been reported to stimulate neutrophils in the intestinal epithelium and increase interleukin-22 expression, which induces activation of the innate immune system and production of antibacterial proteins, such as antimicrobial peptides. Therefore, collectively, these data suggest that SAA3 is involved in the defense against bacterial infection in mouse mammary epithelium.

## 1. Introduction

**Figure 1:**
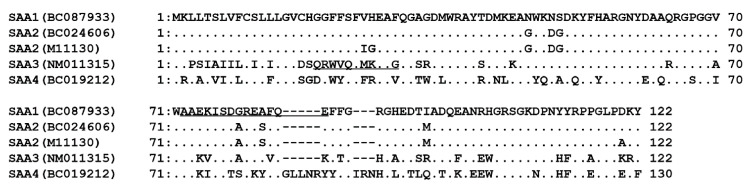
Alignment of amino acid sequences of mouse serum amyloid A (SAA) isoforms. The sequences (AAEKISDGREAFOE and QRWVQFMKEAG) of synthetic SAA1 and SAA3 peptides for immunization to rabbits are underlined. Accession numbers are shown in parentheses.

Systemic amyloid A (AA) amyloidosis is a severe complication of inflammation associated with life-threatening diseases such as rheumatoid arthritis, Crohn’s disease, and gout in humans, but it is also observed in other mammalian species and in birds [1,2]. This disease is caused by the conformational conversion of the circulating soluble precursor protein, serum amyloid A (SAA), into insoluble amyloid fibrils [3]. These fibrils accumulate extracellularly and form deposits in various organs, resulting in fatal organ dysfunction [2]. Chronically high concentrations of blood SAA facilitate the conversion into AA amyloid fibrils [2]. SAA is secreted as an acute phase reactant by the liver under the transcriptional control of interleukin-1 (IL-1) and IL-6 [2]. Blood SAA level increases up to 1000-fold following inflammatory stimulation, and this property makes SAA a biomarker of inflammation, injury, and cancers in humans and animals [4,5]. Based on the amino acid sequence, four SAA isoforms, SAA1-4, have been distinguished in mice. SAA1 and SAA2 genes and proteins (GenBank accession nos. BC087933 and M11130) share 95.1% and 92.6% sequence identities in 396 nucleotides and 122 amino acids, respectively. On the other hand, nucleotide and amino acid sequence identities between SAA1 and SAA3 (NM011315) are 74.3% and 64.7%, respectively (Figure 1). From these sequence differences, it can be concluded that SAA1 and SAA3 have different physiological functions. SAA1 and SAA2 are predominantly produced in the liver as acute phase proteins; they cause formation of AA amyloid fibrils and can be used as biomarkers of inflammation [2,6]. In contrast, SAA3 is mainly expressed in extrahepatic tissues, such as the intestines, the adipose tissue, and the lung, and it does not contribute to the overall SAA level in blood [6,7,8,9]. Previous studies using SAA3 knockout mice demonstrated that SAA3 regulated normal lung development and was required for metabolic function including normal weight [10,11], and SAA3 protects epithelium against acute injuries in colitis [12] and in the lung infected with *Pseudomonas aeruginosa* [13] through induction of neutrophils. In mouse lung, SAA3 stimulates toll-like receptor (TLR) 4 and causes inflammation-like state in early pulmonary metastasis [14]. Mouse SAA3 positively correlates with increased cellular maturation of osteocytes and controls bone development and homeostasis [15]. In vitro studies also reported that *Saa3* mRNA expression was strongly induced by bacterial antigens in mouse colonic epithelial CMT-93 cells [9,16], type-II alveolar epithelial MLE-15 and T7 cells [17], and pulmonary epithelial Club (Clara) C22 cells [18]. In contrast, *Saa1* mRNA expression was not induced by bacterial antigens in any of the mouse epithelial cells, even though *Saa1* mRNA expression was detectable in those cells [9,16,17,18]. Furthermore, the recombinant SAA3 protein up-regulated *Mucin 2* mRNA expression in CMT-93 cells [16,19]. Mucin is a component of the mucus layer, which acts as an epithelial barrier to pathogens [20]. It has been also reported that the presence of SAA3 reduces the severity of non-bacterial infectious colitis [12]. These findings suggest that SAA3 plays a role in the prevention of bacterial infections and in the inflammation during local immunity reactions in mouse epithelium.

However, the relationship between SAA3 expression and local immunity in mouse mammary epithelial cells has not yet been completely elucidated. The purpose of this study was to clarify if SAA3 expression could be up-regulated by the stimuli resembling bacterial infection in mouse mammary epithelial cells. Here, quantitative real-time polymerase chain reaction (PCR), indirect immunofluorescence assay (IFA), and Western blotting (WB) were performed to investigate changes in the expression levels of SAA3 mRNA and protein in mouse mammary epithelial cells after stimulation with bacterial antigens.

## 2. Materials and Methods

### 2.1. Cells

Normal murine mammary gland (NMuMG) epithelial cells were purchased from the European Collection of Authenticated Cell Cultures General Cell Collection, Public Health England (94081121, Salisbury, UK). The cells were maintained in Dulbecco’s modified Eagle’s minimum essential medium (DMEM, #044-29765, Wako, Osaka, Japan) supplemented with 10% fetal bovine serum (FBS) (#A15-701, lot. A70109-0524, PAA Laboratories, Pasching, Austria) and 10 µg/mL insulin (#I0516, Sigma-Aldrich, St. Louis, MO, USA) in a collagen-coated dish (#NCO430167, Corning, Corning, NY, USA).

### 2.2. Treatment with Lipopolysaccharide (LPS) and Lipoteichoic Acid (LTA) for mRNA Expression Analysis

NMuMG cells were seeded at a density of 4×10^5^ cells/well in 6-well collagen-coated plates (#NCO3506, Corning, Corning, NY, USA) and incubated for 15 ± 1 h before experiments. After incubation, the cells were rinsed with sterile phosphate-buffered saline (PBS) and treated with LPS from *Escherichia coli* O111:B4 (#115K4029, Sigma-Aldrich, St. Louis, MO, USA) or lipoteichoic acid (LTA) from *Bacillus subtilis* (L3265, Sigma-Aldrich), which are outer membrane proteins of the Gram-negative and Gram-positive bacteria, respectively. Because our previous studies indicated significantly different responses in cultured mouse colonic, type-II alveolar, and pulmonary epithelial cells [16,17,18], LPS and LTA were diluted to 10 µg/mL in DMEM without FBS. NMuMG cells treated with LPS or LTA were incubated for 2 h at 37 °C in a 5% CO_2_ atmosphere.

### 2.3. RNA Extraction, cDNA Synthesis, and Quantitative Real-Time PCR

For RNA extraction, LPS or LTA-treated cells were collected from the plates by cell lifters. RNA was extracted from the cells immediately using an RNeasy Mini Kit (#74106, Qiagen, Hilden, Germany) following the manufacturer’s instructions. Extracted RNA was quantified using a NanoDropLite spectrophotometer (Thermo Fisher Scientific, Wilmington, DE, USA) and stored at −80 °C until use. Contaminating DNA was eliminated by DNase I (#18068-015, Invitrogen, Carlsbad, CA, USA) treatment, and cDNA was synthesized as described previously [17]. Synthesized cDNA was used for quantitative real-time PCR analysis by using a StepOnePlus thermal cycler (Applied Biosystems, Foster City, CA, USA) as described previously [17].

To evaluate mRNA expression of the *Saa1*, *Saa3*, and glyceraldehyde-3-phosphate dehydrogenase (*Gapdh*) genes, their specific primers were used for quantitative real-time PCR (Table 1). mRNA expression was normalized to that of the reference gene *Gapdh*, which was reported to be a suitable reference gene in NMuMG cells [21], and fold-changes relative to control levels were determined by the ΔΔCT method [22]. For the verification of specific amplification, a melting-curve analysis of amplification products was performed at the end of each PCR reaction. All experiments were replicated at least three times, independently.

### 2.4. Production of Anti-SAA1 Rabbit Serum

An anti-SAA1 serum was produced in a rabbit by immunization with a synthetic SAA1 peptide (AAEKISDGREAFOE), which was generated by Eurofins Genomics (Tokyo, Japan) (Figure 1). An anti-SAA3 rabbit serum generated previously [18] was also used in this study.

### 2.5. IFA (Indirect Immunofluorescence Assay)

To examine SAA1 and SAA3 protein expression levels in cells stimulated with LPS and LTA, IFA was carried out. NMuMG cells were cultured on glass coverslips (#S2441, Matsunami Glass, Kishiwada, Japan) in 6-well plates (#140675, Thermo Fisher Scientific) and incubated for 15 ± 1 h before experiments. LPS and LTA were dissolved to a concentration of 10 µg/mL in DMEM with 2% FBS and added to the cells. After incubation for 4, 8, and 12 h at 37 °C, the cells were washed with PBS, incubated in 800 µL of cold 100% methanol for 5 min, and dried. Then, the cells were blocked with 5% non-fat dried skim milk in PBS for 30 min at room temperature. Anti-SAA1 or anti-SAA3 rabbit serum [18] was used as the first antibody. The cells were incubated with the antibody diluted in PBS (1:50) containing 2% FBS in a humidified chamber for 1 h at room temperature and then washed with PBS. Then, the cells were incubated with the secondary antibody, FITC-goat anti-rabbit IgG (H+L) (1:50, ZYMED Laboratories, South San Francisco, CA, USA) in a humidified chamber for 1 h at room temperature. After washing with PBS, the coverslip was mounted with a drop of 50% glycerol in PBS on a glass slide and sealed with nail polish. The cells were examined with an epifluorescence microscope (ECLIPSE 80i, Nikon, Tokyo, Japan), and the images were captured with ACT-1C software for the DXM 1200C camera (Nikon). The fluorescence of the cells was measured in five or more visual fields randomly selected by using ImageJ (National Institutes of Health, Bethesda, MD, USA). It was normalized to the fluorescence of each respective control and fold-changes relative to control levels were determined.

### 2.6. WB (Western Blotting)

To examine SAA1 and SAA3 protein expression levels in cell supernatants stimulated with LPS and LTA, WB analysis was carried out. NMuMG cells were cultured in the same manner as mentioned above for the analysis of mRNA expression levels. LPS and LTA were dissolved to a concentration of 10 µg/mL in DMEM with 2% FBS and added to the cells. After incubation for 0, 6, 12, and 24 h at 37 °C, 1.0 mL of cell supernatant was collected and centrifuged at 20,000× g for 60 min at 4 °C using an MX-301 centrifuge (Tomy, Tokyo, Japan). Sample precipitates were dissolved in sodium dodecyl sulfate (SDS) sample buffer (0.125 M Tris-HCl, pH 6.8, 10% 2-mercaptoethanol, 4% SDS, 10% glycerol, and 0.01% bromophenol blue) and boiled for 5 min before electrophoresis. These samples were loaded onto a 10% or 12.5% SDS-polyacrylamide gel and electrophoresed. The proteins were transferred onto a polyvinylidene difluoride membrane (Immobilon-P, Millipore, Cork, Ireland), blocked with 5% nonfat milk in Tris-buffered saline (0.01 M Tris-HCl (pH 8.0) and 0.15 M NaCl) containing 0.1% Tween-20 (TBST), and incubated for 30 min at room temperature. Subsequently, the membrane was incubated with the primary anti-mouse SAA1 rabbit polyclonal antibody (1:500, #PAA885Mu01, Cloud-Clone, Houston, TX, USA) or anti-SAA3 rat monoclonal antibody [JOR110A] (1:50, #ab231680, Abcam, Cambridge, UK) diluted with 1% nonfat dried skim milk in TBST for 1 h at room temperature. After washing three times with TBST, the membrane was incubated with anti-rabbit IgG, HRP-linked antibody (1:2000, #7074, Cell Signaling Technology, Danvers, MA, USA) or anti-rat IgG, HRP-linked species-specific whole antibody from goat (1:2000, NA935, GE Healthcare, Buckinghamshire, UK) diluted with TBST for 1 h at room temperature. After washing three times, the peroxidase activity was detected using a Pierce ECL Plus Western Blotting Substrate (Thermo Fisher Scientific) and visualized by a LAS4000mini UV imager (Fujifilm, Tokyo, Japan). Recombinant murine SAA1 (rSAA1) and rSAA3 [19] were used as antigens for positive controls.

### 2.7. Statistical Analysis

Data were collected from at least three independent experiments and expressed as the mean ± standard deviation. Data were analyzed for statistical significance by one-way analysis of variance and the Tukey’s post hoc test.

## 3. Results

### 3.1. Relative Saa1 and Saa3 mRNA Expression Levels

**Figure 2:**
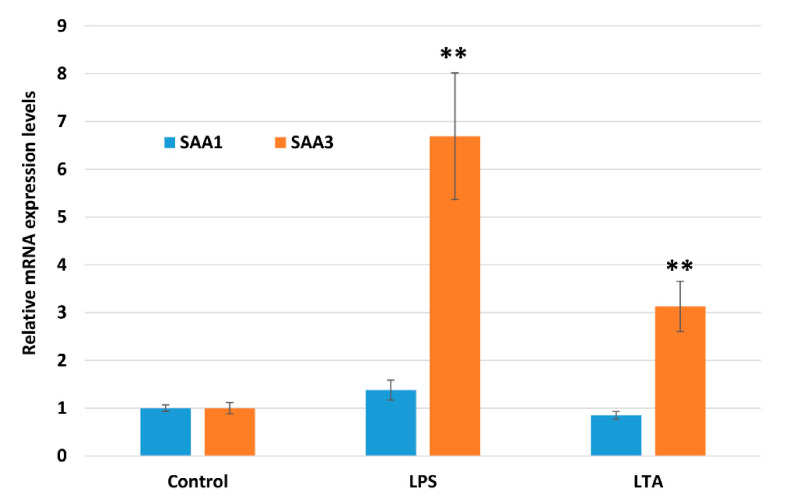
Comparison of Saa1 and Saa3 mRNA expression levels in NMuMG cells treated with lipopolysaccharide (LPS) and lipoteichoic acid (LTA). Data are presented as the mean ± standard deviation of four independent experiments. Asterisks indicate significant difference compared with control levels: ** *p* < 0.01. NMuMG, normal murine mammary gland.

Saa3 mRNA expression levels were significantly increased ~6.5- and ~3-fold by LPS and LTA, respectively, after stimulation for 2 h (Figure 2). In contrast, Saa1 mRNA expression levels were not significantly altered by either LPS or LTA stimulation.

### 3.2. SAA1 and SAA3 Protein Expression Levels

**Figure 3:**
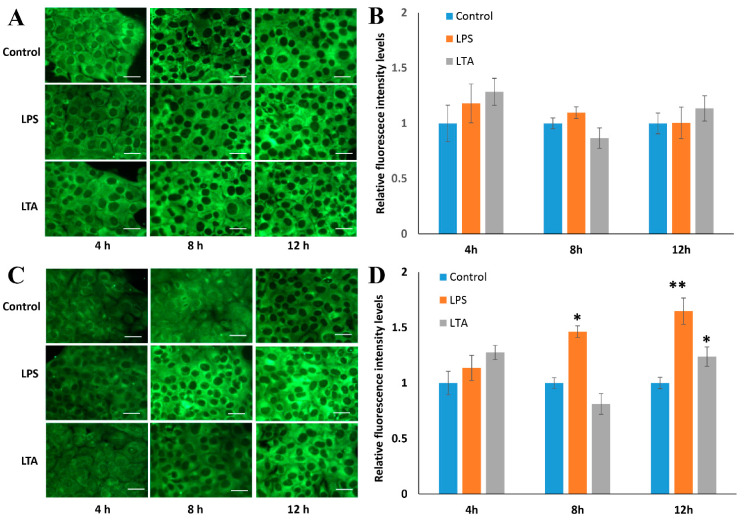
Comparison of SAA1 (A and B) and SAA3 (C and D) protein expression levels in NMuMG cells treated with lipopolysaccharide (LPS) or lipoteichoic acid (LTA) by immunofluorescence analysis (IFA). (**A**,**C**) Fields of view where fluorescence intensity was measured. Scale bar = 20 µm. (**B**,**D**) The relative SAA1 and SAA3 protein expression levels in NMuMG cells following stimulation with LPS or LTA were normalized to those in untreated, control cells. Data are presented as the mean fluorescence of five or more random locations with vertical bars representing standard deviation. Asterisks indicate significant difference compared with control levels: * *p* < 0.05, ** *p* < 0.01. NMuMG, normal murine mammary gland.

**Figure 4:**
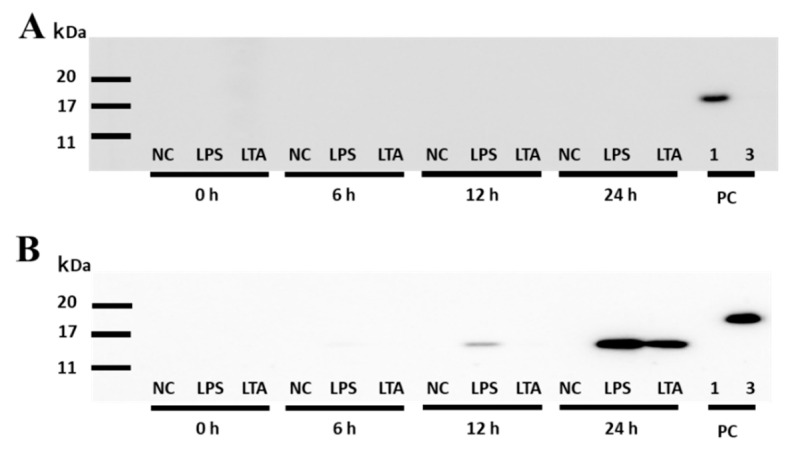
Detection of SAA1 and SAA3 protein expression in NMuMG cell supernatants treated with lipopolysaccharide (LPS) or lipoteichoic acid (LTA) by Western blot. (**A**) Detection of SAA1 in the cell supernatant. Membranes were incubated with the primary anti-mouse SAA1 antibody (dilution of 1:500, 60 s exposure time). (**B**) Detection of SAA3 in the cell supernatant. Membranes were incubated with the primary anti-mouse SAA3 antibody (dilution of 1:50, 120 s exposure time). PC, positive control; 1, recombinant murine SAA1 (rSAA1); 3, rSAA3; NMuMG, normal murine mammary gland.

In IFA, after a 4 h stimulation, there was no significant increase in the fluorescence associated with SAA1 or SAA3 (Figure 3). The SAA1 fluorescence intensity signal did not change significantly at any other time point of the experiment with LPS stimulation (8 h and 12 h; Figure 3A,B). However, the fluorescence intensity of the SAA3 protein increased significantly ~1.4- and 1.6-fold after LPS stimulation for 8 and 12 h, respectively (Figure 3C,D). Furthermore, after 12 h stimulation with LTA, the fluorescence intensity of the SAA3 signal also significantly increased 1.2-fold (Figure 3D). In the WB experiments, no expression of the SAA1 protein was observed after stimulations with LPS or LTA (Figure 4A). SAA3 protein expression was observed after 6 h stimulation with LPS, and it increased further after 12 h and 24 h stimulations (Figure 4B). In addition, SAA3 expression was observed after 24 h LTA stimulation.

## 4. Discussion

Eckhardt et al. demonstrated that *Saa1/2* mRNA was detected in the colon and ileum epithelium of conventionally raised mice and expression of *Saa3* mRNA overlapped *in vivo* [23]. *Saa3* mRNA expression, strongly increased, but not *Saa1* mRNA, by LPS in mouse colonic epithelial CMT-93 cells [23]. In this study, we revealed that LPS and LTA induced *Saa3* mRNA and protein expression in mouse mammary epithelium. Our findings are in accordance with previous studies that demonstrated up-regulation of *Saa3* mRNA expression by bacterial antigens in mouse epithelial cells, such as colonic epithelial CMT-93 cells [16,23,24], type-II alveolar epithelial MLE–15 and T7 cells [17], and pulmonary mucosal epithelial Club (Clara) C22 cells [18].

The quantitative real-time PCR analysis showed that *Saa3* mRNA expression was significantly enhanced in NMuMG cells treated with LPS. Although LTA also enhanced *Saa3* mRNA expression, its effect was weaker than that of LPS stimulation. In contrast, *Saa1* mRNA expression was not significantly changed by either treatment. These results suggest the existence of bacterial antigen, similar to LPS, which can induce *Saa3* mRNA expression in mouse mammary epithelial cells. However, *Saa1* mRNA is unlikely to be induced by such bacterial antigen. This result is consistent with previous studies of the effects of LPS and LTA on *Saa3* mRNA expression levels in epithelial cell lines including CMT93, MLE-15, T7, and C22 cells, although *Saa1* mRNA was detectable and expressed constantly [16,17,18,23]. It is highly likely that the properties of the *Saa3* gene are similar in various mouse mucosal epithelial cells. Besides, Burvenich et al. reported that the bovine mammary gland was highly sensitive to a low dose of LPS from *E. coli* [24]. Therefore, it is likely that mouse mammary epithelium is also highly sensitive to LPS stimulation.

In IFA and WB analysis, LPS induced SAA3 protein expression more strongly than LTA. In contrast, SAA1 protein expression did not change upon the stimulation with LPS or LTA compared to the level in control cells, which was in agreement with the results of real-time PCR. These results indicate that NMuMG responded to the stimulation with LPS cells by up-regulating *Saa3* rather than *Saa1* mRNA and protein expression levels. Considering the results of real-time PCR, it can be concluded that SAA3 protein expression was induced by bacterial antigen stimulation.

In mouse colonic epithelial CMT-93 cells, *Saa3* mRNA expression was induced strongly by the inoculation with *E. coli*, but not with *Staphylococcus aureus* [16]. Moreover, in mouse pulmonary mucosal epithelial C22 cells, the induction of *Saa3* mRNA expression was stronger after LPS than after LTA treatment [18]. These observations suggest that the local immune system of mammary epithelial cells is more sensitive to LPS stimulation than to LTA stimulation, and this differential sensitivity resembles that in other epithelial cells. In this study, we used cultured cells that fail its native characteristics of polarity of the mammary epithelium. Further investigations are required for identification of interaction between bacterial infection and SAA3 expression in mouse mammary epithelium in vivo. In addition, previous studies suggested that mouse SAA3 may play a role in lung development [10], obesity and immunometabolic homeostasis [11], bone homeostasis [15], and metastasis [14,25], as well as in local immunity in a wide variety of epithelium [12,13,16,17,18]. For deep understanding of SAA3 in physiology and pathology in hosts, multifunctional roles of SAA3 should be defined.

In bovine mastitis, the isolated pathogens from the infected area vary, depending on the study areas location and age. For example, Riekerink et al. [26] showed that the main pathogens in the tissue affected by bovine mastitis were *S. aureus* (10.3%), *E. coli* (8.4%), and *S. uberis* (6.3%). Oliveira et al., [27] detected *E. coli* (22.5%), streptococci (12.8%), and *Klebsiella spp.* (6.9%); whereas Verbeke et al. [28] showed the presence of *S. uberis* (18.2%) and *E. coli* (15.5%). Among them, Oliveira et al. [27] have reported a positive correlation between cases with systemic severe signs of illness, such as depression, anorexia, dehydration, or fever, and *E. coli* infection. In that study, it was suspected that the immune system of the mammary epithelium was more sensitive to LPS from Gram-negative bacteria than to LTA from Gram-positive bacteria. Up-regulation of bovine SAA3 expression from mammary epithelial cells were stimulated with LPS, and LTA [29,30,31]. It has been reported that the aggravation of mastitis associated with the presence of *E. coli* is not caused by the bacteria themselves, but by LPS and inflammatory cytokines of the host [32,33]. One of the major mediators involved in the endotoxic shock that aggravates mastitis is tumor necrosis factor-α (TNF-α), and this inflammatory cytokine is down-regulated by interferon-γ [33]. An in vivo experiment in cows demonstrated that all animals in the prophylactic group, which received IFN-γ before the experimental *E. coli* challenge, survived acute *E. coli* mastitis. On the other hand, in the untreated group, many cows developed clinical mastitis and dead from endotoxemia [33]. Ather et al. [10] showed that CD4^+^ T cells from the *Saa3* knockout mouse exhibited increased levels of IL-17A and decreased levels of IL-5, IL-10, IL-13, and IFN-γ. It is possible that the presence of SAA3 protects the host from the endotoxic shock caused by acute *E. coli* mastitis, because SAA3 increases the expression of INF-γ and suppresses the expression of TNF-α and local production of SAA3 by colonic epithelial cells enhanced neutrophilic IL-22 production and, bactericidal activity, and reduced apoptosis [12]. Furthermore, previous studies suggested that bovine SAA3 is also associated with involution and remodeling of the mammary gland [34,35,36]. It is likely that SAA3 plays such functional roles not only in the mammary gland epithelium of cattle but also in that of mice. 

## 5. Conclusions

This study suggests that SAA3 expression is up-regulated in the mammary epithelium in response to the exposure to bacterial antigens. SAA3 may play a role in the local defense against bacterial infections, especially Gram-negative bacteria more strongly than Gram-positive bacteria, in mouse mammary epithelium. 

## Figures and Tables

**Table 1:** Oligonucleotide primers used in quantitative real-time PCR.

Primer	Primer Length	Sequence (5′-3′)	Reference
SAA1/2 F	23 mer	CTGCCTGCCAAATACTGAGAGTC	[5]
SAA1/2 R	25 mer	CCACTTCCAAGTTCCTGTTTATTAC	
SAA3 F	23 mer	GCTGGCCTGCCTAAAAGATACTG	[5]
SAA3 R	24 mer	GCATTTCACAAGTATTTATTCAGC	
GAPDH F	19 mer	TGCACCACCAACTGCTTAG	[17]
GAPDH R	19 mer	GGATGCAGGGATGATGTTC	

SAA, serum amyloid A; GAPDH, glyceraldehyde-3-phosphate dehydrogenase.

## Data Availability

Data are available on request the corresponding author.
